# Dietary Sodium Nitrate Activates Antioxidant and Mitochondrial Dynamics Genes after Moderate Intensity Acute Exercise in Metabolic Syndrome Patients

**DOI:** 10.3390/jcm10122618

**Published:** 2021-06-14

**Authors:** Miguel D. Ferrer, Xavier Capó, Clara Reynés, Magdalena Quetglas, Eduardo Salaberry, Federica Tonolo, Rafael Suau, Bartolomé Marí, Josep A. Tur, Antoni Sureda, Antoni Pons

**Affiliations:** 1 Research Group on Community Nutrition and Oxidative Stress, Health Research Institute of the Balearic Islands (IdISBa), 07120 Palma, Spain; xavier.capo@uib.es (X.C.); clara.reynes@uib.es (C.R.); m.quetglas@uib.es (M.Q.); eduardo.salaberry@uib.es (E.S.); pep.tur@uib.es (J.A.T.); antoni.sureda@uib.es (A.S.); antonipons@uib.es (A.P.); 2 Research Group on Community Nutrition and Oxidative Stress, IUNICS, and Laboratory of Physical Activity Sciences, University of the Balearic Islands, 07122 Palma, Spain; federica.tonolo@phd.unipd.it; 3 CIBEROBN (Physiopathology of Obesity and Nutrition CB12/03/30038), Carlos III Health Institute, 28029 Madrid, Spain; 4 Department of Biomedical Sciences, University of Padova, 35131 Padova, Italy; 5 Sports Medicine Service, Consell Insular de Mallorca, 07010 Palma, Spain; rsuau@conselldemallorca.net (R.S.); bmari@conselldemallorca.net (B.M.)

**Keywords:** exercise, inflammation, metabolic syndrome, nitrate, oxidative stress, supplementation

## Abstract

Exercise can induce a pro-inflammatory response in aged subjects with metabolic disorders and nitrate supplementation has shown anti-inflammatory effects. We evaluated the influence of dietary nitrate on the response of the antioxidant and mitochondrial dynamics genes to acute exercise in peripheral blood mononuclear cells (PBMCs), as well as the antioxidant and the inflammatory response of PBMCs against immune stimulation. Metabolic syndrome patients participated in a crossover study in which they consumed a beverage containing 16 mM sodium nitrate or a placebo with the same composition without nitrate before performing a submaximal test at 60–70% of their maximal heart rate for 30 min. The intake of nitrate increased the nitrate plus nitrite plasma levels about 8-fold and induced the upregulation of catalase, superoxide dismutase, glutathione peroxidase, mitofusin 2 and PGC1α in PBMCs after exercise. The gene expression of catalase and TNFα was enhanced by phorbol myristate acetate (PMA) only in the placebo group, while the glutathione peroxidase expression was enhanced by PMA only after nitrate intake. The intake of nitrate by metabolic syndrome patients induces an antioxidant and mitochondrial response to exercise at the same time that it attenuates the pro-inflammatory response to immune stimulation.

## 1. Introduction

Inorganic dietary nitrate elicits beneficial physiological effects in the reversion of certain features of the metabolic syndrome, including a reduction in blood pressure, vasodilatory improvements in blood flow, and anti-coagulant, antioxidant and anti-inflammatory effects [1,2,3]. Dietary nitrate may have therapeutic utility against obesity and associated metabolic complications, possibly by increasing adipocyte mitochondrial respiration and by dampening inflammation and oxidative stress [3]. In fact, animal, human and epidemiological studies have shown that dietary nitrate and nitrite modulate inflammatory processes and immune cell function and phenotypes [4,5,6,7]. The current evidence on the efficacy of nitrate-rich plant foods and of other sources of nitrate and nitrite suggests that immune cells and immune-vasculature interactions are important targets of dietary nitrate interventions [3]. Dietary nitrate increases the expression of anti-inflammatory markers in visceral fat and macrophages, and this is associated with the reduction of NADPH oxidase-derived superoxide production in macrophages, enhancement of adipocyte mitochondrial respiration, increased protein expression of total mitochondrial complexes and elevated gene expression of uncoupling protein (UCP)-1 in mice with metabolic disfunctions. The actions of oral nitrate are mediated through the nitrate–nitrite–nitric oxide signaling pathway [8], resulting from the cooperation between oral and intestinal microbiota and the nitrite reductase activity of heme-proteins, xanthine oxidase, cytochrome c oxidase and other oxidases [9]. However, it is also critical to consider potential unwanted and adverse effects of these compounds in human studies. The potential health risks associated with the intake of nitrosamines, nitrate and nitrite in connection with endogenous nitrosation reactions have been evaluated [10]. It was concluded that there is inadequate evidence in experimental animals for the carcinogenicity of nitrate [11], while the International Agency for Research on Cancer (IARC) reviewed the carcinogenicity of nitrite and concluded that there is limited evidence in experimental animals for the carcinogenicity of nitrite per se [11].

The practice of regular physical activity appears to be one of the main contributors to the prevention of chronic diseases and it is considered as a health promoter [12]. Several studies describe functional changes in immune cells, such as peripheral blood mononuclear cells (PBMCs), that could contribute to reduce the chronic inflammation and oxidative stress [12,13,14]. In fact, elderly people with a more active lifestyle exhibit increased antioxidant machinery and an attenuated age-associated inflammatory status in PBMCs [14]. Regular daily activity and an active lifestyle leads to a scenario with enhanced oxidative metabolism capabilities, in which the antioxidant defenses are also enhanced to cope with subsequent stressors. Similarly, the practice of regular exercise counteracts the signs of chronic inflammation by lowering interleukin (IL)-6 circulating levels and the toll-like receptor (TLR)2 protein levels in PBMCs, while the expression of the anti-inflammatory IL-10 is enhanced in active subjects [13]. Although acute exercise may act as an inducer of antioxidant and anti-inflammatory status in healthy young sportsmen [12], it has the opposite effect on metabolic syndrome patients. Acute exercise of moderate intensity and duration increases the plasma levels of the pro-inflammatory cytokines tumor necrosis factor α (TNFα) and intercellular adhesion molecule 1 (ICAM1), of prostaglandin E1 (PGE1) and prostaglandin E2 (PGE2) and of 16 hydroxyl-palmitate (16HPAL) in metabolic syndrome patients [6], pointing to a mild pro-inflammatory response of metabolic syndrome patients to acute exercise. This pro-inflammatory response, which can be related to an increased production of reactive oxygen species during the exercise session, is attenuated by the intake of sodium nitrate 30 min before exercise [6], which avoids the enhancing effects of acute exercise on pro-inflammatory cytokine production and on prostaglandins and other oxylipins plasma levels, while reducing the capabilities of PBMCs and neutrophils to produce these oxylipins.

Chronic exposure to endotoxins elicits intestinal inflammation and impairs the gut barrier function, potentially resulting in systemic inflammation with elevated concentrations of biomarkers associated with metabolic syndrome [15]. Metabolic endotoxemia initiates low-grade chronic inflammation in metabolic syndrome patients and provokes the progression towards more advanced cardiometabolic disorders [16]. Lipopolysaccharide (LPS) is an endotoxin derived from intestinal bacteria. Only small amounts of LPS translocate through the gut in healthy individuals [17,18,19], thus preventing the activation of TLR4, which would lead to the release of inflammatory cytokines and the installation of a chronic inflammation status [20]. Regular and acute physical exercise enhance PBMC activation after LPS stimulation, increasing cytokine production via TLR signaling pathways, and also enhancing antioxidant enzyme defenses [21,22,23]. Our aim was to assess the antioxidant and mitochondrial responses to acute exercise in metabolic syndrome patients and to determine if the intake of nitrate before the exercise would modulate these responses. We hypothesized that oral nitrate influences the response of the antioxidant and mitochondrial dynamics genes to acute exercise in PBMCs, as well as the antioxidant and the pro-/anti-inflammatory response of PBMCs against the immune stimulation with the endotoxins LPS and phorbol myristate acetate (PMA).

## 2. Materials and Methods

### 2.1. Study Participants

A total of 15 male participants with metabolic syndrome were enrolled in this study. The sample size was calculated taking into account the relative expression of genes and proteins (expressed as a percentage) and using the formula *n* = 2 × [(Z_α_ + Z_β_)^2^ × σ^2^]/∆^2^ [24], considering an α error of 0.05, a β error of 0.10, an estimated standard deviation (σ) of 20, and a minimum difference to be detected (∆) of 25, which resulted in an *n* of 13 participants. The variables chosen were the relative expressions of inflammatory genes and proteins. Inclusion criteria included men aged 55–75 years old, with a body mass index (BMI) between 27 and 40 kg/m^2^, that met at least three criteria for metabolic syndrome according to the updated harmonized criteria. Exclusion criteria were: (a) active cancer or a history of malignant tumors; (b) documented history of previous cardiovascular disease; (c) impossibility to follow a recommended diet or to carry out physical activity; (d) inability or unwillingness to give informed consent. The study protocol was in accordance with the Declaration of Helsinki, and all procedures were approved by the Research Ethics Committee of the Balearic Islands (reference no. 3560/17 PI).

### 2.2. Anthropometric and Clinical Measurements

We measured height with a mobile anthropometer (Kawe 44444, Asperg, Germany), and weight was measured with a digital scale (Tefal, sc9210, Rumilly, France). Body mass index (BMI, kg/m^2^) was calculated using the previous measures of weight and height. Waist and hip circumference (cm) were measured using a non-stretch measuring tape (Kawe 43972, Kirchner and Wilhelm GmBH Co., KG, Asperg, Germany). To avoid inter-observer variation, all anthropometric measurements were performed by one observer. Blood pressure was measured in triplicate with the participant seated after 5 min of resting, using a validated semi-automatic oscillometer (Omron HEM-705CP, Kyoto, Japan) [25].

Venous blood samples were extracted from the antecubital vein of participants in vacutainers containing ethylenediaminetetraacetic (EDTA) as anticoagulant. Glucose, cholesterol and triglycerides were determinate using an autoanalyzer (Technicon DAX System, Technicon Instruments Corp., Tarrytoen, NY, USA).

### 2.3. Experimental Procedure

**Figure 1:**
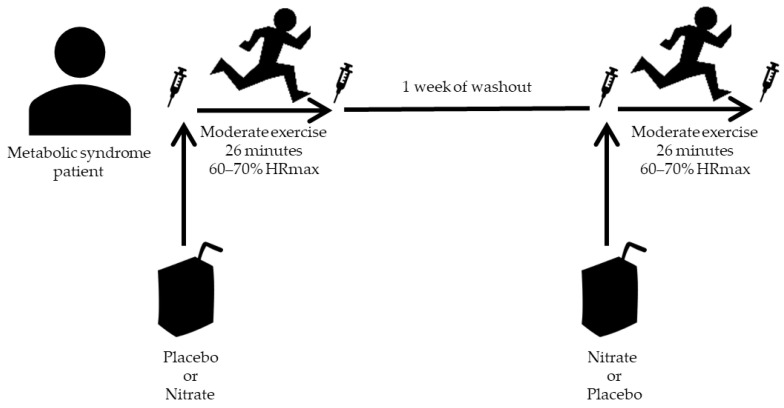
Schematic design of the randomized, double-blind, 2 × 2 crossover study. Participants that took the placebo beverage in the first experience took the nitrate-rich beverage in the second one, and vice versa.

The participants were cited at the laboratory the first time in the morning in two different occasions, placed one week apart, to perform a submaximal exercise test. In the first visit, the participants were randomized 1:1 either to the placebo or the supplemented beverages. On each occasion, all the participants had the same standardized breakfast (around 500 kcal, 60% carbohydrates, 25% lipids, 15% proteins), avoiding nitrate-rich foods, 1 h before the first blood extraction. The recommendations for this standardized breakfast were 50 g of bread with 15 g of olive oil and 30 g of cooked ham, with 200 mL of skimmed milk and 200 mL of fruit juice. All the participants were specifically instructed not to alter their pattern of physical activity between the first exercise test and the second one, so every subject could act as their own control with the only difference being the composition of the drink they took before the test. After pre-exercise blood sampling, the oral nitrate-reducing capability was determined in each subject (see below). Then, the participants drank 500 mL of one of two almond-based beverages: placebo (made up of 3.0% almond, 0.8% sucrose, 0.6% olive oil and 1.8 mg/100 mL alpha-tocopherol acetate) (Vitalmend SL, Palma, Spain), which was used as a vehicle beverage in previous studies with sportsmen [26,27], or the same beverage supplemented with sodium nitrate (16 mM) in a double-blind 2 × 2 crossover fashion. That is, the participants took their assigned beverage (placebo or nitrate) in the first visit and the opposite beverage (nitrate or placebo) in the second visit (as displayed in Figure 1), meaning that at the end of the study each participant had taken the two beverages and could act as their own control. After 30 min of each beverage consumption, at around 10 a.m., the participants performed a submaximal test at 60–70% of their maximal heart rate (HRmax), calculated using Tanaka’s equation (HRmax = 208 − 0.7 × Age) [28], on a motorized treadmill (H/P/cosmos^®^, pulsar^®^). The total duration of the test was 30 min, during which the first 4 min were used to progressively increase the speed of the treadmill until reaching 4 km/h, which corresponded to 60–70% of HRmax, followed by 26 min at this constant speed. Figure 1 shows a scheme of this study design. O_2_ uptake (VO_2_) and CO_2_ production (VCO_2_) were recorded constantly with a computerized metabolic cart (Ultima Series^®^, Medgraphics cardiorespiratory diagnostics, Saint Paul, MN, USA). The energy expenditure and the energy efficiency parameters were calculated during the 26 min at 60–70% of HRmax. Finally, a second venous blood extraction was performed 30 min after the end of the exercise. Only 8 out of 15 participants performed and completed the two exercise tests with their blood samples available at the required timing. Reasons for participants dropping out during the study included alterations of heart rate during the test, rescinding permission to obtain blood samples just before or after the test and inability to perform the second test.

### 2.4. Plasma and PBMCs Isolation

Blood was centrifugated at 900× *g* for 30 min at 4 °C to obtain plasma and samples were stored at −80 °C until their utilization.

PBMCs were isolated following a method previously described [26]. We carefully introduced blood on Ficoll^®^Paque PLUS in a proportion of 1.5:1 (v:v), and samples were centrifuged at 900× *g* at 4 °C for 30 min. After centrifugation, the PBMCs layer was collected and transferred to a new tube. PBMCs fraction was washed twice with phosphate buffered saline (PBS) and finally centrifuged for 10 min at 1000× *g* at 4 °C. The cellular pellet was resuspended in Tripure (Roche Diagnostics, Mannheim, Germany) for RNA extraction.

### 2.5. ‘Ex Vivo’ Stimulation of PBMCs with LPS and PMA

PBMCs were isolated from 2 mL of blood of participants in pre- and post-exercise conditions after the intake of placebo or nitrate-enriched beverages as described above. Isolated cells were then resuspended and cultured for 2 h at 37 °C in 2 mL of RPMI 1640 culture medium containing 2 mM L-glutamine in three different conditions: (1) addition of 1 µg/mL of LPS; (2) addition of 10 ng/mL of PMA; (3) control conditions without stimulant addition. Immune cells concentration on culture media was therefore the same as in blood. At the end of the 2 h samples were centrifuged at 900× *g* for 5 min at 4 °C and the cellular pellet was resuspended in Tripure (Roche Diagnostics, Mannheim, Germany) for RNA extraction.

### 2.6. Oral Nitrate-Reducing Capability and Measurement of Nitrite and Nitrate Concentrations

The participants first held 10 mL of water in their mouth for 2 min. After the mouth rinse was collected, the participants held 10 mL of a solution of 8 mM sodium nitrate. We collected each mouth rinse into a sterile tube and centrifuged it at 4500 rpm at 4 °C for 10 min. The supernatant was collected and stored at −80 °C.

Nitrite and nitrate concentrations in plasma and mouth rinse supernatants were measured using a NO analyzer (NOA 280i; Sievers, GE Power and Water, Boulder, CO, USA), detecting the chemiluminescence produced by the reaction between ozone and nitric oxide. To obtain these measurements, plasma samples were deproteinized with zinc sulphate 4% and 0.4 M NaOH to avoid scum interferences during the determination [29]. Mouth rinse supernatant samples were analyzed directly without deproteinization.

Nitrite was transformed into nitric oxide in a customized glass purge vessel infused with nitrogen using 3.5 mL of KI reagent, 13.5 mL of glacial acetic acid and 200 µL of anti-foaming reagent. Nitrate and nitrite were transformed into nitric oxide in the glass purge vessel using 12 mL of VCl_3_ diluted in HCl 1M and 200 µL of anti-foaming. The purge vessel was further connected to the NO analyzer. Nitrate and nitrite standard curves were constructed to determine the concentration of nitrate and nitrite in plasma and mouth rinse supernatants.

### 2.7. Western Blot

UCP3, manganese superoxide dismutase (MnSOD), cytochrome c oxidase subunit IV (CoxIV), mitofusin 1 (Mfn1) and mitofusin 2 (Mfn2) protein levels were determined by Western blot. PBMC samples were lysed with 250 µL of RIPA buffer (250 mM Tris/HCl, pH 8.0, 4.4% NaCl, 5% IGEPAL^®^, 2.5% deoxycholic acid, 0.5% sodium dodecyl sulfate, (SDS)). Proteins of total cell extract were separated by size using SDS polyacrylamide electrophoresis and transferred onto a nitrocellulose membrane using Trans-Blot Turbo Transfer System (Bio-Rad Laboratories, Hercules, CA, USA). The membranes were incubated overnight at 4 °C with primary monoclonal antibodies: anti-UCP3 antibody (1:1000) (Chemicon International, Temecula, CA, USA), anti-Mn-SOD antibody (1:1000) (Calbiochem, San Diego, CA, USA), anti-CoxIV (1:500), (Millipore, Billerica, MA, USA), anti-Mfn1 (1:200) and anti-Mfn2 (1:1000) antibodies (Santa Cruz Biotechnology, Santa Cruz, CA, USA) and were then incubated with a secondary peroxidase-conjugated antibody. Protein bands were visualized by Immun-Star^®^ Western C^®^ Kit reagent (Bio-Rad Laboratories, Hercules, CA, USA). The chemiluminescence signal was captured with a Chemidoc XRS densitometer (Bio-Rad Laboratories, Hercules, CA, USA) and analyzed with Quantity One-1D Software (Bio-Rad Laboratories, Hercules, CA, USA). We used Precision Plus Protein TM Kaleidoscope TM (Bio-Rad Laboratories, Hercules, CA, USA) as a molecular weight marker, and the band density of each protein was quantified according to the loading control (actin), used as housekeeping.

### 2.8. PBMCs RNA Extraction and Real-Time PCR Assay

The mRNA expression of the genes detailed in Supplementary Table S1 was determined by real time polymerase chain reaction (RT-PCR) with human 18S ribosomal as the reference gene. Total RNA was purified from PBMCs by Tripure extraction (Roche Diagnostics, Mannheim, Germany) following a procedure previously described [30]. The isolated RNA (1 µg) from each subject was reversely transcribed using Expand Reverse Transcriptase (50 U) (Roche Diagnostics, Mannheim, Germany) for 60 min at 42 °C and 5 min at 99 °C in a final volume of 10 µL, according to the manufacturer’s instructions. The resulting cDNA (3 µL) was amplified and quantified using the LightCycler^®^ 480 instrument (Roche Diagnostics, Mannheim, Germany) with FastStart DNA MasterPLUS SYBR Green I kit (Roche Diagnostics, Mannheim, Germany). The primers used and the amplification conditions are shown in Table S1. mRNA levels of the placebo group before the exercise test were arbitrarily referred to as 1.

### 2.9. Statistical Analysis

Statistical analysis was carried out using Statistical Package for Social Sciences (SPSS v.21.0 for Windows). The normal distribution of the data was confirmed using the Shapiro–Wilk test. A paired sample t-test was applied when comparing two groups. A two-way ANOVA was applied when analyzing the effect of two factors (nitrate supplementation and exercise or nitrate supplementation and immune stimulation). When significant effects or interactions were found, a one-way ANOVA was applied as post-hoc to determine the differences between the groups involved. Results are shown as mean ± standard error of the mean (SEM), and *p* < 0.05 was considered statistically significant.

## 3. Results

Aged, obese or overweighed adults, or those with hypertension, hyperglycemia or triglyceridemia participated in the study. Subject characteristics, as well as basal blood markers, are presented in Table 1.

The participants performed two controlled acute exercise tests at 60–70% of their maximal heart rate. They drank a placebo beverage before performing one exercise test and a nitrate rich beverage before performing the other exercise test. Table 2 shows the characteristics of the exercise test and the indicators of energy expenditure and efficiency. The exercise duration and power were similar in both exercise tests; however, the oxygen consumption rate, both expressed as mL/min or mL/min Kg, was significantly lower after the intake of the nitrate rich beverage than after the intake of the placebo beverage. Similarly, both total energy expenditure and energy expenditure rate of the exercise test were significantly lower after the intake of the nitrate-rich beverage than after the intake of the placebo beverage. Energy efficiency of the exercise test, expressed as the kcal consumed to run 1 km at a speed of 4 km/h, was also significantly lower after the nitrate-rich beverage intake than after the placebo. The use of lipids or carbohydrates to obtain energy to perform the exercise test was not influenced by the intake of the beverages, as indicated by the maintained values of the respiratory quotient. Therefore, the intake of the nitrate-rich beverage 30 min before the acute exercise reduced the oxygen cost of exercise, but without influencing the energy source (lipids or carbohydrates) used to sustain the exercise.

The participants exhibited an oral capability to reduce nitrate to nitrite in a few minutes. The participants washed their oral cavity using water or a nitrate solution for 2 min. The nitrate and nitrite levels in these oral washing liquids are also shown in Table 2. Both nitrite and nitrate concentrations were significantly higher after oral washing with nitrate solution than with water.

**Figure 2:**
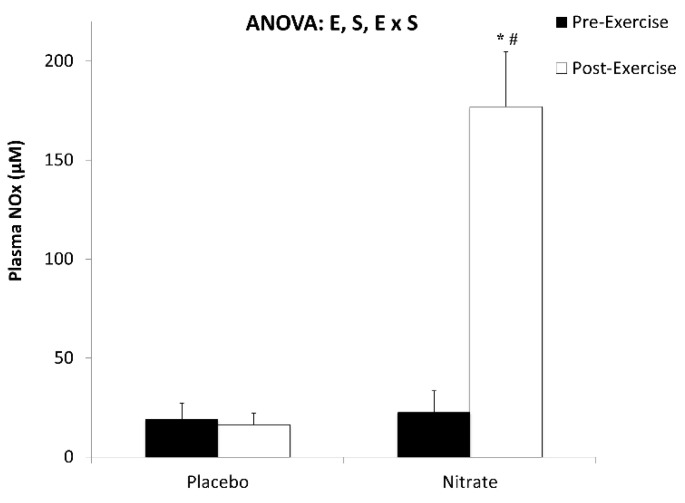
Effects of exercise and oral nitrate supplementation on nitrite and nitrate (NO_x_) plasma levels from metabolic syndrome patients. Results represent mean ± SEM. Statistical analysis: two-way ANOVA. Letters indicate significant effects of exercise (E) or supplementation (S) or significant interaction of the two ANOVA factors (E x S). * Significant differences with respect to placebo; (#) significant differences with respect to pre-exercise, *p* < 0.05.

The intake of the nitrate-rich beverage 30 min before performing the exercise test significantly increased about 8-fold the nitrate plus nitrite (NOx) levels in plasma 30 min after exercising (Figure 2). Therefore, plasma nitric oxide precursors were significantly more available for the muscle during exercise after the intake of the nitrate-rich beverage.

**Figure 3:**
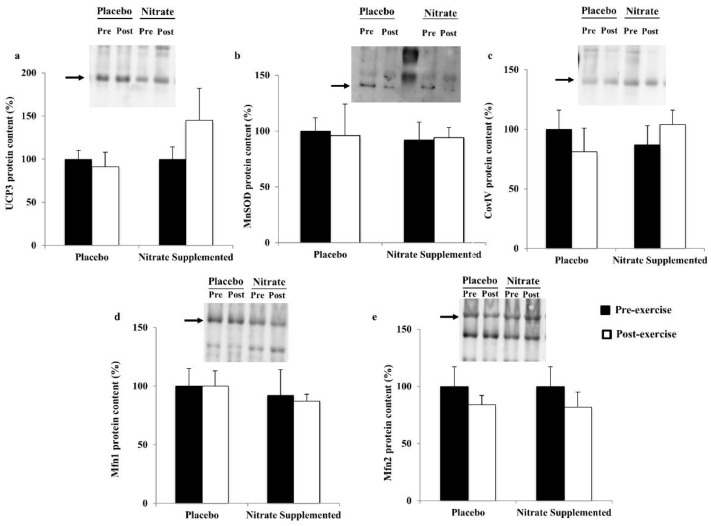
Effects of exercise and nitrate intake on mitochondrial and antioxidant protein levels in PBMCs from metabolic syndrome patients: (**a**) Uncoupling protein 3 (UCP3); (**b**) manganese superoxide dismutase (MnSOD); (**c**) cytochrome c oxidase subunit IV (CoxIV); (**d**) mitofusin 1 (Mfn1); (**e**) mitofusin 2 (Mfn2). Results represent mean ± SEM. Statistical analysis: two-way ANOVA. No significant effects were found, *p* < 0.05.

The effects of acute exercise and nitrate supplementation on the antioxidant, respiratory chain complex and mitochondrial dynamics protein levels in PBMCs are shown in Figure 3. Neither acute exercise nor nitrate-rich beverage intake influenced the MnSOD, UCP3, Mfn1, Mfn2 and CoxIV protein levels in PBMCs 30 min after ending acute exercise.

The effects of acute exercise and oral nitrate supplementation on the expression of antioxidant genes and genes related with mitochondrial dynamics in PBMCs are shown in Table 3.

The basal expression of mitochondrial NADH dehydrogenase subunit 5 (MitoND5), UCP3 and heme oxygenase 1 (HO1) were maintained after exercise independently of the previous intake of nitrate-rich or placebo beverages. The antioxidant enzymes gene expression was significantly enhanced by the interaction of exercise and nitrate supplementation. Acute exercise significantly increased the catalase, MnSOD and glutathione peroxidase (GPx) gene expression when performed after the nitrate-rich beverage intake but not when performed after the placebo beverage intake. In fact, the gene expression of catalase, MnSOD and GPx after exercise was significantly higher after the nitrate-rich beverage intake than after the placebo beverage intake. Similarly, the expression of Mfn2 and peroxisome proliferator-activated receptor alpha coactivator (PGC1α) followed a similar pattern to the expression of antioxidant enzymes, with increased expression after acute exercise only in the nitrate supplemented individuals. On the contrary, the expression of Mfn1 significantly decreased after acute exercise after the nitrate-rich beverage intake, while it was maintained at the basal level after the placebo beverage intake. Acute exercise significantly decreased the mitochondrial transcription factor A (Tfam) expression independently of the beverage consumption. The nuclear respiratory factor 2 (Nrf2) was significantly influenced by the nitrate-rich beverage intake, which avoided the down-regulation associated with exercise.

We finally analyzed ‘ex vivo’ the effects of nitrate dietary supplementation on the antioxidant and pro-inflammatory response induced by LPS or PMA in PBMCs. PBMCs isolated after nitrate-rich or placebo beverage intake were incubated for 2 h in the presence of LPS or PMA or in the absence of these two immunomodulators (control culture).

The effects of PMA inducing the gene expression of catalase, GPx and TNFα in PBMCs were significantly different to those induced by LPS, and they were also dependent on the type of beverage consumed (Table 4). The incubation of PBMCs for 2 h in the presence of LPS did not influence the gene expression of catalase, GPx and TNFα, neither after placebo nor nitrate-rich beverage intake. However, the incubation of PBMCs for 2 h with PMA enhanced the expression of catalase, GPx and TNFα, depending on the type of beverage consumed. The gene expression of both catalase and TNFα was enhanced by PMA in the PBMCs isolated after placebo beverage intake, but the intake of nitrate-rich beverage avoided these enhancing effects of PMA. Inversely, the GPx expression was enhanced by PMA in the PBMCs isolated after nitrate-rich beverage intake, while it was maintained at the control level in the PBMCs isolated after placebo intake. The IL6 gene expression in PBMCs was significantly influenced by the presence of LPS or PMA, but no significant differences between groups were observed.

## 4. Discussion

Regular physical activity prescription is a key point for healthy aging and chronic disease management and prevention. An active lifestyle and daily activities enhance the antioxidant defenses and oxidative metabolism capabilities in PBMCs from healthy elderly [14,31,32,33,34]. The ROS produced at moderate levels during acute exercise may have signaling roles that can modulate muscle contraction, and antioxidant and mitochondrial status of PBMCs and neutrophils, even exerting antioxidant protection on the organism [35,36,37]. Acute exercise enhances mitochondrial biosynthesis, fission and fusion processes in PBMCs of young sportsmen [12,38,39], while training stimulates not only the biogenesis of mitochondria, but also the removal of old and unhealthy mitochondria in skeletal muscle [40]. Old subjects with metabolic syndrome exhibit a higher degree of oxidative stress and a proinflammatory state than old healthy subjects of the same age and gender [41]. The present study shows that acute exercise performed in a cyclergometer by metabolic syndrome patients at 60%–70% of their maximal heart rate for about 30 min does not influence the levels of mitochondrial proteins related with antioxidant (UCP3, MnSOD), respiratory (CoxIV) or mitochondrial remodeling (Mfn1, Mfn2) functions. However, this acute exercise performed by metabolic syndrome patients reduces the gene expression of CoxIV and Tfam and maintains the basal expression of MitoND5, UCP3, HO1, MnSOD, GPx, CAT, Mfn1, Mfn2, Nrf2 and PGC1α. These results suggest that the acute exercise performed by metabolic syndrome patients might reduce the mitochondrial respiratory capabilities (CoxIV) and mitochondrial biosynthesis (Tfam). These effects of acute exercise performed by metabolic syndrome patients are in contrast with those described after a more intense and longer exercise session performed by young well-trained sportsmen [12], in which acute exercise increased the protein levels of UCP2 and Mfn2 and the expression of CoxIV and PGC1α. These contrasting results reinforce the fact that the age and physical and training status or pathologic situation of the subjects markedly affect the metabolic and molecular response to a given exercise challenge and point to the idea that acute exercise may worsen the oxidative and mitochondrial status of PBMCs in metabolic syndrome patients. This response is in accordance with previous results of the effects of acute exercise performed by metabolic syndrome patients on plasma oxidative and pro-inflammatory markers [6]. Acute exercise increases the plasma concentration of TNFα, intercellular adhesion molecule (ICAM1), and products of saturated and polyunsaturated fatty acid peroxidation, such as PGE1, PGE2 and 16-hydroxypalmitate. These potentially harmful effects of acute exercise on the oxidative and inflammatory status of metabolic syndrome subjects were prevented by the intake of a nitrate-rich beverage before exercise. In this instance, it was reported that nitrate intake prevents the enhancing effects of acute exercise on the plasma concentration of TNFα, ICAM1, PGE1, PGE2 and 16-HPAL, while it reduces the capabilities of PBMCs and neutrophils to produce oxylipins [6].

It has been suggested that dietary nitrate may have therapeutic utility against obesity and its associated metabolic complications, possibly by increasing mitochondrial respiration, mitochondrial dynamics and by dampening inflammation and oxidative stress [3]. We evidence that nitrate consumption influences the changes in the expression of antioxidant genes and genes related to the mitochondrial dynamics in response to acute exercise. Acute exercise performed after the intake of a nitrate-rich beverage enhances the expression of MnSOD, GPx, CAT, Mfn2 and PGC1α, and reduces the expression of Mfn1 and Tfam, while these responses were not observed when the exercise was performed without having consumed the nitrate-rich beverage (except for Tfam). Therefore, the combination of acute exercise and nitrate intake improves the antioxidant capabilities through enhancing MnSOD, GPx and CAT gene expression and it may also act as an inducer of mitochondrial remodeling through Mfn2 and PGC1α gene expression. Mfn2 plays a major role in mitochondrial fusion and in the maintenance of mitochondria-endoplasmic reticulum contact sites [42,43,44]. Mfn2, but not Mfn1, is required for the adaptation of mitochondrial respiration to stress conditions and for the production of reactive oxygen species (ROS) upon pro-inflammatory activation [45]. The PBMCs response to acute exercise performed by metabolic syndrome patients after the intake of the nitrate-rich beverage is therefore similar to the response observed after acute exercise performed by young well-trained sportsmen [12]. These effects of acute exercise on antioxidant and mitochondrial remodeling capabilities by nitrate intake are probably mediated through the nitrate–nitrite–nitric oxide signaling pathway [46]. This pathway for nitric oxide generation results from the cooperation between oral and intestinal microbiota and the nitrite or nitrate reductase enzyme pseudo-activities of heme-proteins, xanthine oxidase, cytochrome c oxidase and others oxidases [47].

Nitrate intake before exercise reduces the oxygen cost of exercise both in sportsmen and in metabolic syndrome subjects [6,48,49,50]. In the present study, we confirm the reduction of oxygen cost of exercise performed by metabolic syndrome patients who consumed the nitrate-rich beverage, without influencing the energy source (lipids or carbohydrates) used to sustain the exercise, and the operative nitrate–nitrite–nitric oxide pathway through the reduction of nitrate to nitrite in the oral cavity and the increased plasma levels of nitrate and nitrite after acute exercise. It has been described that nitrate increases mitochondrial efficiency in humans as it increases the P/O ratio (the amount of ATP produced/oxygen used) [50]. In fact, the mitochondrial respiratory chain produces nitric oxide from nitrite through the acceptance of electrons in the complexes I and III [51]. This mitochondrial nitric oxide production contributes to the reduction of the oxygen cost of ATP production. The mitochondrial use of nitrite in place of oxygen additionally reduces the mitochondrial production of ROS, because the reduction of nitrite to nitric oxide does not produce ROS.

Mitochondrial ROS are in a state of dynamic balance between production and elimination [52]. Many studies have found that dietary nitrate therapy can decrease the mitochondrial generation of ROS [53,54,55]. Additionally, it has been evidenced that nitrite attenuates the NADPH oxidase-derived superoxide generation in activated macrophages and human monocytes via a nitric oxide-dependent mechanism [56]. It has also been evidenced that xanthine oxidase plays an important role in the production of nitric oxide from nitrite and also from nitrate [57,58]. It has been reported that xanthine oxidase can use nitrite or nitrate (instead of oxygen) to oxidize xanthine to uric acid, thus reducing the oxygen cost and avoiding superoxide anion production.

Antioxidant [6,59] and anti-inflammatory [58,60] effects of oral nitrate have already been pointed out. A mechanistic approach points to nitric oxide as responsible for these antioxidant and anti-inflammatory effects, but we suggest that the low production of ROS can be directly due to nitrite or nitrate, contributing to a decrease in the production of harmful amounts of ROS and enhancing their role as signaling molecules at low concentrations. In this way, the effects of nitrate intake on the induction of the expression of antioxidant enzymes, Mfn2 and PGC1α after exercise could be related with a decreased mitochondrial or xanthine oxidase production of ROS as result of the use of nitrate/nitrite in place of oxygen to generate nitric oxide. We previously evidenced in ‘in vitro’ studies with PBMCs of metabolic syndrome patients that a low rate of H_2_O_2_ production can enhance the expression of mitochondrial dynamics-related proteins such as Mfn2, whereas a high rate of H_2_O_2_ production diminishes the expression of mitochondrial dynamics-related proteins such as Mfn1 and Tfam [61]. This behavior, described for the response of PBMCs to low or high rates of H_2_O_2_ production, is very similar to the response to acute exercise after the intake of a nitrate-rich beverage or a placebo, respectively. Acute exercise at 60–70% of the maximal heart rate performed by metabolic syndrome patients could induce a high rate of H_2_O_2_ production, with damaging effects on the gene expression, but the previous intake of nitrate would reduce H_2_O_2_ production, and low levels could act as signaling molecules for antioxidant and mitochondrial remodeling genes. Although more evidence is required to fully confirm this hypothetical mechanism of action, our results contribute to understanding the mechanisms by which oral nitrate and nitrite show beneficial effects in cardiovascular and metabolic diseases, in addition to the direct role of nitric oxide.

LPS interacts with Toll-like receptors (TLRs) and enhances the NFκB signaling pathway, leading to a low-grade of chronic inflammation that provokes the progression of metabolic syndrome towards more advanced cardiometabolic disorders [62]. PMA, a molecular mimetic of diacylglycerol [63] used to activate immune cells by mimicking their response to acute exercise [64], activates several isoforms of protein kinase C (PKC), particularly PKC*δ* and *ε*, which in turn activate the NFκB signaling pathway [65]. Both LPS and PMA can increase ROS production in immune cells by the assembly and activation of NADPH-oxidases or by the activation of mitochondrial respiratory chain function [30,64,66,67,68]. Moreover, both PMA and LPS increase antioxidant enzyme gene expression in PBMCs [30], neutrophils [69,70] and HL60 cells [64]. The intake of a nitrate-rich beverage influences the antioxidant and pro-inflammatory gene expression profile after the immune stimulation of PBMCs from metabolic syndrome patients with PMA or LPS. The antioxidant and proinflammatory gene expressions are scarcely enhanced by LPS in PBMCs from metabolic syndrome patients, with high inter-individual variation. As the PBMCs are isolated from metabolic syndrome patients with a low grade of chronic inflammation, the low inflammatory response to LPS stimulation could reflect a certain degree of tolerance to this stimulus, possibly at the level of the TLRs. PMA, however, induces a measurable pro-inflammatory response in these immune cells from metabolic syndrome patients. The mechanisms of PMA involve the NFκB signaling pathway, not through the interaction with TLRs but by the activation of PKC. In an ‘ex vivo’ setting, nitrate intake avoids the enhanced expression of CAT and TNFα induced by PMA in PBMCs from metabolic syndrome patients but, on the other hand, it activates the expression of GPx in response to PMA stimulation. These results are in accordance with a role of the oral nitrate–nitrite–nitric oxide pathway reducing the rate of ROS production in PBMCs stimulated by PMA. The pro-inflammatory effects of PMA, such as the induction of cyclooxygenase-2, NFκB, IL8 and TNFα expression, are abolished by the antioxidant vitamin C in ‘ex vivo’ neutrophils from sportsmen [70]. A partial reduction of the ROS production rate in PBMCs because of the nitrate intake could avoid the enhanced expression of CAT and TNFα induced by PMA. In a similar way, the excessive rate of ROS production by PBMCs stimulated by PMA could avoid the activation of GPx, but the reduced ROS production in PBMCs from patients that consumed the nitrate-rich beverage would allow enhancing its expression, as occurs in vivo after the exercise test. Therefore, oral nitrate exerts anti-inflammatory and antioxidant effects against the immune stimulation of PBMCs by PMA and in a lower extension against the immune stimulation by LPS, which is already attenuated in metabolic syndrome patients. The main limitations of this study are that only men were enrolled, there was a limited number of participants and the evaluation only examined the effects of one dose of nitrate and one specific protocol of exercise.

## 5. Conclusions

Our results show that the intake of nitrate by metabolic syndrome patients induces an antioxidant and mitochondrial dynamics response to moderate intensity acute exercise, which is not observed in the absence of nitrate intake, and at the same time attenuates the pro-inflammatory response to immune stimulation.

## Figures and Tables

**Table 1:** Anthropometrical, clinical and nutritional characteristics of the participants.

Anthropometrical Parameters	Mean ± SEM
Age (years)	66.5 ± 2.3
Weight (kg)	80.2 ± 4.2
Height (cm)	166 ± 2.8
Body Mass Index (BMI, kg/m^2^)	29.3 ± 1.7
Waist Circumference (cm)	102 ± 4
Systolic Blood Pressure (mmHg)	141 ± 5.7
Diastolic Blood Pressure (mmHg)	81.3 ± 1.7
**Clinical Parameters**	
Glucose (mg/dL)	93.3 ± 2.8
Triglycerides (mg/dL)	115 ± 7.6
Cholesterol (mg/dL)	174 ± 14
HDL-Cholesterol (mg/dL)	44.3 ± 4.9
LDL-Cholesterol (mg/dL)	106 ± 11

Results represent mean ± SEM (*n* = 8).

**Table 2:** Parameters of an exercise test at 60–70% of maximal heart rate after the intake of a placebo or a nitrate-rich beverage.

	Placebo	Nitrate	Effect Size
Test duration (min)	26.0 ± 0.1	26.0 ± 0.1	0.18
Speed (Km/h)	4.0 ± 0.0	4.0 ± 0.0	
Heart Rate (Beats/min)	108 ± 11	104 ± 9	0.18
VO_2_ (mL/min)	1478 ± 89	1389 ± 83 *	0.46
VO_2_ (mL/Kg min)	18.8 ± 1.5	17.7 ± 1.5 *	0.33
VCO_2_ (mL/min)	1311 ± 66	1275 ± 80	0.22
Energy Expenditure (Kcal/min)	7.3 ± 0.4	6.9 ± 0.4 *	0.42
Total Energy Expenditure (Kcal)	189 ± 10	179 ± 11 *	0.43
Energy Efficiency (Kcal/Km)	109 ± 6.1	103 ± 6.2 *	0.25
Respiratory Quotient	0.89 ± 0.03	0.92 ± 0.01	0.44
Energy from lipids (%)	38.6 ± 12	29.5 ± 2.7	0.49
Oral Washing	Water	Nitrate	
Nitrite (nM)	2718 ± 343	17,970 ± 3943 *	2.22
Nitrate (µM)	17.5 ± 4.21	2488 ± 201 *	7.10

Results represent mean ± SEM. Effect size was calculated using Cohen’s d index. Statistical analysis: paired Student’s *t*-test; * Represents significant differences vs. placebo or water, *p* < 0.05.

**Table 3:** Effects of exercise and nitrate supplementation on mitochondrial and antioxidant gene expression in PBMCs of metabolic syndrome patients.

Gene (%)	Placebo		Nitrate Supplemented		Statistics		
	Pre-Exercise	Post-Exercise	Pre-Exercise	Post-Exercise	Two-Way ANOVA		
					E	S	E x S
MitoND5	1.00 ± 0.15	1.51 ± 0.83	0.97 ± 0.22	1.32 ± 0.46			
CoxIV	1.00 ± 0.14	0.37 ± 0.11 *	0.90 ± 0.20	0.59 ± 0.14	E		
UCP3	1.00 ± 0.24	0.75 ± 0.35	0.60 ± 0.15	0.29 ± 0.09			
HO1	1.00 ± 0.18	0.55 ± 0.17	0.96 ± 0.22	0.71 ± 0.19			
MnSOD	1.00 ± 0.33	0.49 ± 0.20	1.42 ± 0.54	3.72 ± 0.92 ^#^		S	
GPx	1.00 ± 0.34	0.47 ± 0.20	1.14 ± 0.37	38.7 ± 9.6 *^,#^	E	S	E x S
CAT	1.00 ± 0.59	0.46 ± 0.28	1.27 ± 0.44	34.7 ± 7.8 *^,#^	E	S	E x S
Mfn1	1.00 ± 0.26	1.14 ± 0.88	1.06 ± 0.52	0.18 ± 0.04	E		
Mfn2	1.00 ± 0.34	0.34 ± 0.11	1.07 ± 0.33	4.56 ± 1.04 *^,#^	E	S	E x S
Nrf2	1.00 ± 0.14	0.32 ± 0.09	1.16 ± 0.17	1.07 ± 0.37		S	
PGC1α	1.00 ± 0.31	0.59 ± 0.35	1.22 ± 0.39	5.61 ± 1.34 ^#^		S	E x S
Tfam	1.00 ± 0.19	0.24 ± 0.08 *	1.00 ± 0.31	0.27 ± 0.09 *	E		

CAT: catalase; CoxIV: cytochrome c oxidase subunit IV; GPx: glutathione peroxidase; HO1: heme oxygenase 1; IL6: interleukin 6; MitND5: mitochondrial NADH dehydrogenase subunit 5; MnSOD: manganese superoxide dismutase; Mfn1: mitofusin 1; Mfn2: mitofusin 2; Nrf2: nuclear respiratory factor 2; PGC1α: peroxisome proliferator-activated receptor alpha coactivator; Tfam: transcription factor A, mitochondrial; TNFα: tumor necrosis factor Alpha; UCP3: uncoupling protein 3. Results represent mean ± SEM. Statistical analysis: ANOVA. Letters indicates significant effects of exercise (E) or supplementation (S) or significant interaction of the two ANOVA factors (E x S). * Represents significant differences vs. pre-exercise. ^#^ Represents significant differences vs. placebo, *p* < 0.05.

**Table 4:** Effects of dietary nitrate supplementation and ‘in vitro’ immune stimulation with lipopolysaccharide (LPS) or phorbol myristate acetate (PMA) on antioxidant and inflammatory gene expression in PBMCs.

		Control	LPS	PMA	ANOVA		
					M	S	M x S
GPx	Placebo	1.00 ± 0.37 ^a^	3.03 ± 1.78 ^a^	6.53± 2.76 ^a^	M	S	M x S
	Nitrate	1.00 ± 0.26 ^a^	5.30 ± 3.74 ^a^	48.8 ± 35.6 ^b^			
CAT	Placebo	1.00 ± 0.42 ^a^	4.93 ± 2.03 ^a,b^	23.1 ± 11.4 ^b^			M x S
	Nitrate	1.00 ± 0.29 ^a,b^	18.9 ± 10.8 ^a,b^	1.80 ± 0.94 ^a,b^			
IL6	Placebo	1.00 ± 0.39	4.51 ± 1.99	0.46 ± 0.25	M		
	Nitrate	1.00 ± 0.28	7.75 ± 4.63	0.16 ± 0.06			
TNFα	Placebo	1.00 ± 0.36 ^a^	4.28 ± 1.81 ^a,b^	14.6 ± 5.65 ^b^			M x S
	Nitrate	1.00 ± 0.36 ^a,b^	7.40 ± 3.08 ^a,b^	0.78 ± 0.42 ^a,b^			

CAT: catalase; GPx: glutathione peroxidase; HO1: heme oxygenase 1; IL6: interleukin 6; TNFα: tumor necrosis factor Alpha. Results represent mean ± SEM. Statistical analysis: Two-way ANOVA. Letters indicate significant effects of immune stimulation (M) or nitrate supplementation (S) or significant interaction of the two ANOVA factors (M x S). Different letters (^a,b^) indicate significant differences between groups, *p* < 0.05.

## Data Availability

The data presented in this study are available on request from the corresponding author.

## Supplementary Materials

The following are available online at https://www.mdpi.com/article/10.3390/jcm10122618/s1, Table S1: Primer sequence and annealing temperatures used for the real-time PCR.
