# Supplementary material for: Dietary Sodium Nitrate Activates Antioxidant and Mitochondrial Dynamics Genes after Moderate Intensity Acute Exercise in Metabolic Syndrome Patients
Source: J Clin Med. 2021 Jun 14;10(12):2618. doi: 10.3390/jcm10122618 (PMC8232343; doi:10.3390/jcm10122618)
Supplement: Supplementary file 1 [file jcm-10-02618-s001.zip › jcm-1235979-supplementary.pdf]

Supplementary Table 1. Primer sequence and annealing temperatures used for the real-time PCR.

Gene	Accession Numbe		Primers	Temperature (°C)
**18S**	NR_003286	Fw:<br>Rv:	5′-GAC TCA ACA CGG GAA ACC CTC AC-3'<br>5′-GAC TCA ACA CGG GAA ACC CTC AC-3'	60
**MitND5**	YP_003024 036	Fw:<br>Rv:	5′-CGG CTG AGA GGG CGT AGG-3′<br>5′-GAT GAA ACC GAT ATC CGG CCGA-3′	60
**CoxIV**	NM_00186 1	Fw:<br>Rv:	5′-AGA AGC ACT ATG TGT ACG GCCC-3′<br>5′-GGT TCA CCT TCA TGT CCA GCAT-3′	60
**UCP3**	NM_00335 6	Fw:<br>Rv:	5′-CGT GGT GAT GTT CAT AAC CTA TG-3′<br>5′-CGG TGA TTC CCG TAA CAT CTG-3′	60
**HO1**	NM_00213 3	Fw:<br>Rv:	5′-CCA GCG GGC CAG CAA CAA AGT GC-3'<br>5′-AAG CCT TCA GTG CCC ACG GTA AGG-3'	60
**MnSOD**	NM_00132 281	Fw:<br>Rv:	5′-GAG AGG TAC CAG GAG GCG TTG-3'<br>5′-CAA GCC AAC CCC AAC CTG AGC-3'	60
**GPx**	NM_00058 1	Fw:<br>Rv:	5′-TTC CCG TGC AAC CAG TTT G-3'<br>5′-TTC ACC TCG CAC TTC TCG AA-3'	60
**CAT**	NM_00175 2	Fw:<br>Rv:	5′-TTT GGC TAC TTT GAG GTC AC-3'<br>5′-TCC CCA TTT GCA TTA ACC AG-3'	60
**Mfn1**	NM_03354 0	Fw:<br>Rv:	5′-TGT TTT GGT CGC AAA CTC TG-3′<br>5′-CTG TCT GCG TAC GTC TTC CA-3′	60
**Mfn2**	NM_01487 4	Fw:<br>Rv:	5′-ATG CAT CCC CAC TTA AGC AC-3′<br>5′-CCA GAG GGC AGA ACT TTG TC-3′	60
**Nrf2**	NM_00616 4	Fw:<br>Rv:	5′-GCG ACG GAA AGA GTA TGA GC-3'<br>5′-GTT GGC AGA TCC ACT GGT TT-3'	60
**PGC1α**	NM_00133 0751	Fw:<br>Rv:	5′-CAC TTA CAA GCC AAA CCA ACA ACT-3′<br>5′-CAA TAG TCT TGT TCT CAA ATG GGG A-3′	62
**Tfam**	NM_00320 1	Fw:<br>Rv:	5′-CAA GAC AGA TGA AAC CAC CTC-3'<br>5′-AGA TTG GGG TCG GGT CAC T-3'	60
**IL6**	NM_00060 0	Fw:<br>Rv:	5′-TAC ATC CTC GAC GGC ATC TC-3'<br>5′-ACT CAT CTG CAC AGC TCT GG-3'	63
**TNFα**	NM_00059 4	Fw:<br>Rv:	5′-CCC AGG CAG TCA GAT CAT CTT CTC GGA A-3'<br>5′-CTG GTT ATC TCT CAG CTC CAC GCCA TT-3'	59

18S: ribosomal 18S; CAT: catalase; CoxIV: cytochrome c oxidase subunit IV; GPx: glutathione peroxidase; HO1: heme oxygenase 1; IL6: interleukin 6; MitND5: mitochondrial NADH dehydrogenase subunit 5; MnSOD: manganese superoxide dismutase; Mfn1: mitofusin 1; Mfn2: mitofusin 2; Nrf2: nuclear respiratory factor 2; PGC1α: peroxisome proliferator-activated receptor alpha coactivator; Tfam: transcription factor A, mitochondrial; TNFα: tumor necrosis factor Alpha; UCP3: uncoupling protein 3.
